# Perspectives on Kidney Disease Education and Recommendations for Improvement Among Latinx Patients Receiving Emergency-Only Hemodialysis

**DOI:** 10.1001/jamanetworkopen.2021.24658

**Published:** 2021-09-09

**Authors:** Tessa K. Novick, Santiago Diaz, Francisco Barrios, Doris Cubas, Kavyaa Choudhary, Paul Nader, Raymonda ElKhoury, Lilia Cervantes, Elizabeth A. Jacobs

**Affiliations:** 1 Division of Nephrology, University of Texas at Austin, Dell Medical School, Austin; 2 Department of Internal Medicine, University of Texas at Austin, Dell Medical School, Austin; 3 The University of Texas at Austin, Austin; 4 Division of General Internal Medicine and Hospital Medicine, University of Colorado, Boulder; 5 Maine Medical Center Research Institute, Scarborough

## Abstract

**Importance:**

In most states, undocumented Latinx immigrants with kidney failure receive dialysis in acute care settings on an emergency-only basis. How much kidney disease education Latinx immigrants receive and how to improve kidney disease education and outreach among Latinx populations are unknown.

**Objective:**

To understand the kidney disease educational gaps of Latinx individuals who need but lack access to scheduled outpatient dialysis.

**Design, Setting, and Participants:**

This qualitative study used semistructured interviews in a Texas hospital system from March 2020 to January 2021 with 15 individuals who received emergency-only dialysis when they were first diagnosed with kidney failure. Demographic information was collected, and a thematic analysis was performed using the constant comparative method on interviews after they were audio-recorded, translated, and transcribed verbatim. Data analysis was performed from April 2020 to February 2021.

**Main Outcomes and Measures:**

Subthemes and themes from semistructured interviews.

**Results:**

All 15 persons interviewed (9 male individuals [60%]; mean [SD] age, 51 [17] years) identified as Hispanic, 11 (73%) were born in Mexico, and none reported knowing about their kidney disease more than 6 months before starting dialysis. The themes identified were (1) lack of kidney disease awareness, (2) education provided was incomplete and poor quality, (3) lack of culturally concordant communication and care, (4) elements that Latinx patients receiving emergency-only dialysis want in their education, (5) facilitators of patient activation and coping, and (6) Latinx patient recommendations to improve community outreach.

**Conclusions and Relevance:**

Latinx adults receiving emergency-only dialysis are usually unaware of their kidney disease until shortly before or after they start dialysis, and the education they receive is poor quality and often not culturally tailored. Participants made feasible recommendations on how to improve education and outreach among Latinx communities.

## Key Points

**Question:**

What are the perspectives of Latinx individuals receiving emergency-only hemodialysis on the kidney disease education they received, and what recommendations do these individuals offer on how to improve education and outreach among Latinx populations?

**Findings:**

In this qualitative study including 15 Latinx patients who received emergency-only hemodialysis, participants were unaware of their kidney disease until shortly before or after they started dialysis, and the education they received was poor quality and not culturally tailored. Potential improvements in kidney disease education and outreach were identified.

**Meaning:**

These findings suggest that Latinx populations may receive substandard education about their kidney disease and that education and community outreach efforts for Latinx populations could be improved.

## Teaser

This qualitative study examines perspectives on and possible improvements for kidney disease education among Latinx patients receiving emergency-only hemodialysis.

## Introduction

People with kidney failure who lack resources for scheduled outpatient dialysis receive treatment in acute care settings on an emergency-only basis in most states. They present to hospitals with life-threatening electrolyte abnormalities, volume overload, or symptoms of uremia, and are admitted for dialysis. Compared with scheduled outpatient dialysis, emergency-only dialysis is associated with 5-fold higher 1-year mortality and 14-fold higher 5-year mortality,^1,2^ worse morbidity and quality of life,^3,4^ and higher acute care utilization and health care costs.^1^

Most individuals who receive emergency-only dialysis are undocumented immigrants, and the majority of undocumented immigrants are from Latin America.^5,6^ There are an estimated 5500 to 8857 undocumented immigrants with kidney failure in the United States.^7,8^ Latinx individuals who receive emergency-only dialysis also frequently lack primary care and pre–kidney failure nephrology care and report limited English-language proficiency.^9,10,11,12,13,14^ They often have suboptimal control of diabetes and hypertension, low use of recommended medications, and low chronic kidney disease awareness, leading to a 50% higher risk of kidney failure.^9,14^

It is unclear what education Latinx immigrants with kidney failure receive about kidney disease because of fragmented care. Patient perspectives on how to improve kidney disease education and outreach within this population have not been studied. Our objective was to explore the perspectives of Latinx individuals receiving emergency-only hemodialysis on the kidney disease education they received prior to the onset of kidney failure and obtain patient recommendations on how to improve education and outreach among Latinx communities.

## Methods

### Study Design

We conducted 15 semistructured interviews with Latinx adults with kidney failure who relied on emergency-only dialysis. All interviews were conducted one-on-one in Spanish over the phone from March 2020 to January 2021. The consent form was read in Spanish by the interviewer. Participants provided verbal consent and received a $20 gift card to a local grocery store. We followed the Standards for Reporting Qualitative Research (SRQR) reporting guideline for reporting our findings.^15^ The study was approved by the University of Texas at Austin, Dell Medical School institutional review board.

### Setting and Participants

Participants were eligible if they were aged 18 years or older, reported speaking Spanish as their primary language, and relied on emergency-only dialysis at 2 hospitals in Austin, Texas, affiliated with the Dell Medical School. Participants were not asked about their immigration status. The majority of participants initially had a tunneled catheter for vascular access. To receive dialysis, patients had to be critically ill, defined as the presence of uremic symptoms, volume overload, or electrolyte abnormalities. Patients usually stayed in the hospital overnight for 2 consecutive hemodialysis sessions, were then discharged, and repeated the cycle 1 to 2 weeks later.

Participants were recruited using convenience sampling. The research team was notified when individuals presented for emergency-only dialysis, and patients meeting eligibility criteria were invited to participate. The invitation to participate occurred over the phone after the individual had received at least 1 dialysis session to ensure they had mental capacity. Collection of demographic information and the semistructured interview occurred over the phone following hospital discharge. Race and ethnicity data were self-reported.

### Interview Guide

We conducted semistructured interviews using open-ended questions. We asked participants to describe how they found out about their kidney disease, the education they received, what would have helped them and their families understand their diagnosis and dialysis, and suggestions for improving patient education and outreach among Latinx communities (Box).

Box. **Interview Guide**

1. Following completion of informed consent, inform the participant that there are no right/wrong answers, the answers they provide will not be shared, this information will not affect their medical care, and it is okay to skip any questions.
2. Begin with questions that seek to understand how the participant learned about their kidney disease, what education they received, and what they wished they knew before starting dialysis:
  - First, tell me the story of when you first found out about your kidney problems. (Probes: What was that like? How did you respond? How did your family respond? What was your understanding of your treatment options?)
  - If you had a friend who was starting dialysis and wanted to know what to expect, what would you tell them? (Probe: What else do you know now that you wish you knew when you first started dialysis?)
3. Next explore the participants’ family/caregivers’ understanding of their kidney disease and the dialysis process.
  - With regard to your kidney problem, what does your family understand about what is happening to your body right now?
  - In your opinion, what would help them better understand what you are experiencing?
4. Finally, explore the participant’s recommendations regarding the best way to improve outreach and kidney disease education in their community.
  - Studies have shown that people in our community are at a higher risk for needing dialysis than other populations. What do you think would help our community understand more about kidney health?
  - If doctors wanted to teach our community how to keep their kidneys healthy, what would be the best way to teach them? (Probe: Where would be the best place to do this?)

### Data Collection

Three Spanish-speaking members of the research team conducted the semistructured interviews (S.D., D.C., and F.B.). Interviews were audio recorded, translated, transcribed verbatim, and deidentified (by Datagain [Secaucus, NJ]). The interviewers reviewed each transcript for accuracy. Recruitment ceased when no new perspectives or themes emerged from the data. Participants were asked about demographic information, including age, sex, ethnicity, birthplace, employment and education information, information on etiology of kidney disease and treatment history, household information, family history of kidney disease, and information on health-related social needs.^16^

### Statistical Analysis

Two members of the research team (T.N., D.C., and K.C.) independently coded interview transcripts. Regular meetings were held to reach consensus on codes, subthemes, and themes. Transcribed interviews were analyzed using the constant comparative method in order to generate theory that was close to the data.^17^ Data analysis was performed from April 2020 to February 2021.

## Results

Among the 15 participants interviewed in this study who had kidney failure and relied on emergency-only dialysis, 15 (100%) identified as Hispanic, 11 (73%) were born in Mexico, and 9 (60%) were male individuals; the mean (SD) age was 51 (17) years; and the mean (SD) time they had been receiving dialysis when interviewed was 9 (17) months (Table 1). None were aware they had kidney disease more than 6 months before starting dialysis, and 7 (47%) were not informed until the day they presented with kidney failure. Health-related social needs were common among participants. We identified 6 themes and 17 subthemes (Table 2).

**Table 1.** Participant Characteristics

Characteristic	Participants, No. (%) (N = 15)
Interview length, mean (SD), min	27 (12)
Age, mean (SD)	51 (17)
Sex	
Male	9 (60)
Female	6 (40)
Identified as Hispanic	15 (100)
Preferred interview in Spanish	15 (100)
Birthplace	
Mexico	11 (73)
Central America (Honduras, El Salvador)	3 (20)
United States	1 (7)
Unemployed	15 (100)
Education level	
No School	3 (20)
Less than high school	7 (47)
High school	4 (27)
More than high school	1 (7)
Health insurance	
None	1 (7)
Local safety-net insurance	9 (60)
Other	5 (33)
Other family member with kidney disease	8 (53)
Cause of kidney disease	
Unknown	4 (27)
Diabetes	8 (53)
Hypertension	2 (13)
Hypertension and diabetes	1 (7)
When informed of kidney disease	
>6 mo before dialysis start	0
0-6 mo before dialysis start	9 (60)
Not informed prior to dialysis start	6 (40)
Months on dialysis, mean (SD)	9 (17)
Frequency of dialysis, presentations/mo, mean (SD)	3 (1.2)
No. of people living at home, mean (SD)	5 (3)
Housing instability^a^	4 (27)
Food insecurity^b^	4 (27)
Utility issues^c^	5 (33)
Transportation issues^d^	2 (13)

^a^ Housing instability: a report of currently having a steady place to live but worried about losing it in the future, or currently not having a steady place to live.

^b^ Food insecurity: a report of sometimes or often not having enough food or money to buy more in the past 12 months.

^c^ Utility issues: a report of having the electric, gas, oil, or water company threaten to shut off services within the past 12 months.

^d^ Transportation issues: a report of lack of reliable transportation keeping them from medical appointments, meetings, work, or getting things needed for daily living in the past 12 months.

**Table 2.** Themes and Subthemes With Illustrative Quotations

Themes and subthemes	Summary and illustrative quotations
Theme 1: Lack of kidney disease awareness	Participants reported most education happened after they started dialysis, and that prior to developing symptoms of kidney failure, they were largely unaware they had kidney disease.
Dialysis was unexpected	“I came into the hospital with shortness of breath, nausea, dizziness, and I could barely really move my hands and feet … the next day the doctors told me that I had end-stage renal failure. It was really shocking to me because nobody had ever said anything about my kidneys before.”
	“I didn’t know I had kidney issues … I started to feel a little fatigued and had quite a bit of coughing. When I entered the hospital, they didn’t let me go. It turned out … I had fluid in my lungs because of my kidneys… the same day they put a catheter in me for dialysis.”
Missed opportunity for prevention	“As [Hispanic people], we don’t understand it, if they say, this is harmful, don’t do it, we don’t understand. Maybe we let things happen and then we have consequences. In the end, you realize that you could have done something about your diet or your nutrition, and you didn’t want to do anything, so now you face the consequences.”
	“I didn’t receive that education where I was told to take care of myself … it hurts us a lot because we’re not educated in the sense of food or anything like that.”
Theme 2: Education provided was incomplete and poor quality	The education provided typically occurred while participants were admitted for dialysis and were acutely ill. It was often fragmented, and learning occurred by piecemeal over time through discussions with clinicians, nursing staff, other patients, and through their own trial and error.
Treatment options influenced by nonmedical factors	“Only dialysis [was] discussed, and they weren’t very clear about a transplant, because I don’t have documents, and since I don’t have that, I can’t enroll in a waiting list. I don’t have insurance to get dialysis. I’m going to get dialysis only when I feel bad.”
	“They didn’t tell me anything … because if I go to the [dialysis] clinic nobody will treat me. They won’t take me in because I don’t have any money or insurance of anything.”
Education not delivered at appropriate level of health literacy	“I don’t even know what else to ask them about. The only thing I’d like to hear from them are some positive answers, right? Like, ‘you’ll recover from this, you will get better, you just need to do this and that …’ I’m not sure if I’m still hurt from the dialysis I got, but I don’t know what they did to me, whether they opened me up.”
	“Since we don’t have any experience … sometimes you can’t even answer correctly … or many people don’t have the capacity of answering with words … our minds go blank.”
Education provided while experiencing distress	“I don’t really ask much about dialysis. They tell me a lot of things, but it’s not that I don’t care, it’s just that sometimes people talk to me about dialysis, and when I get there, I am in a serious condition, so I am asleep. I sleep all day, so they can talk to me, but it’s as if I ignored them, because they talk to me but I’m not paying attention.”
	“You’re interested in feeling better, not in learning more.”
Theme 3: Lack of culturally concordant communication and care	The education was often provided in another language, was not culturally tailored, and did not incorporate their support system.
Education did not occur in preferred language	“When they talk to us in English … we don't know anything, but we just say yes out of respect. But we don't really know what are the good things and the bad things about what you're telling me.”
	“Most people talk to me in English … I just don’t understand English.”
Education did not incorporate support systems	“I don’t think [my family] knows much …. [They understand that my] kidneys are damaged … and that’s why I need dialysis. And I need to do it to stay alive, more than anything.”
	“They understand this is something related to the kidneys … [but] get worried because instead of improving, [it appears I’m] getting worse.”
Theme 4: Elements Latinx patients receiving emergency-only dialysis want in their education	Participants highlighted things they wished they were told when they started dialysis. To improve clinician messaging, they suggested using language that is direct and culture concordant, using visual aids for complex topics like vascular access, and incorporating traditional foods into dietary counseling.
Give overall prognosis and describe expected symptoms	“I’ve always liked to have people tell me things clearly, directly … instead of saying one thing and then another, and to keep me wrapped around it. I prefer them to tell me how things are, so that I can know if I can have high hopes or not. If the report is serious, okay, then I’ll know.”
	“Explain to them the process of everything, from when you start … that there's going to be a time when they're going to start getting cramps ... and when you finish dialysis … you actually get dizzy and you feel quite exhausted.”
Use culturally concordant language	“[The doctors should] speak in Spanish so I can understand them because sometimes they speak in English and I don't understand it. There are some things I understand and some things I don't understand, but for me, it would be much better in Spanish.”
	“I think it would help me more if it was explained to me by someone who speaks Spanish fluently.”
Use visual aids	“Images have always caught my attention. So, if it was explained to me with images, it would be a lot better …”
	“When I was told that they would put a fistula in my hand … I don’t exactly understand what a fistula is like. I do understand more or less how it works, but I would like to have some images like, look, this is a fistula, it works like this.”
Incorporate traditional foods into dietary counseling	“I don't eat salt, I don't eat tortilla, I don't eat bread. I eat flour tortilla, because they told me in the hospital that's the one I could eat the most. No corn tortilla, no sweet bread. No banana, no potato.”
	“As I’m Hispanic, it’s mostly my nutrition. We don’t eat very healthy; tacos, cakes, we eat whatever, you know? And I have a specific diet.”
Theme 5: Facilitators of patient activation and coping	Encouragement, family, and faith were identified as factors that helped participants cope with the diagnosis of kidney failure and treatment.
Encouragement and normalization of emotional experience	“I would say, ‘It's okay, just relax and keep going.’ Because really the process that [they’re] going to have to go through [is] a big one. And [they’re] going to be very sad because you get the melancholy, you get the feeling that you're not worth anything, that you're not good for anything with this. And then you start to have a lot of doubts ... [they] need a word of encouragement.”
	“It’s been very helpful to me [to have people say] ‘let’s try it out … what we want here is the best for you.’”
Include family in decision-making	“My son is 4 months old, he was born 8 days after I found out I needed dialysis, and it’s the same thing that many people, many friends would have done. Make the effort for your child, make the effort for your family.”
	“I was one of those people that [said] I can’t do it and I don’t want to do it anymore. But if you have family and grandkids and everything like I do, you won’t do it for you, you would do it for them.”
Faith	“You don’t have a normal life the way it was before ... I thank God because he’s giving me another chance to live. Even if I’m sick … I can still fight. You simply get sad … but at the same time I [thank] God for giving me another chance and not dying… I have a lot of faith in God, and I have to push forward.”
	“Because you think the world is closing out on you, and you don’t need to close the doors to the world. Even if they say you will die, you have to trust God and yourself and try to do the impossible to survive.”
Theme 6: Latinx patient recommendations to improve community outreach	Participants acknowledged that knowledge about nutrition, healthy lifestyle behaviors, and kidney health are needed in Latinx communities. To optimize impact, education should include patient testimonials in the form of videos. They recommended using social media, and providing education at churches or other community locations because many do not have access to medical care and do not receive education in clinical settings.
Promote diet and lifestyle education	“I think they should start educating all [Hispanic people] by telling them that they need to walk, exercise, eat healthily. Maybe [offer] more help [by] teaching them about nutrition. Not to stop eating, but to eat healthy. Then in the afternoon, or when you have time, to walk … or to do some exercise … because we lack information.”
	“The best way to teach us is firstly telling us how to eat properly. [Tell people], ‘You need to eat healthy, otherwise you’ll keep getting ill.’ Because there isn’t a more dangerous pandemic than the one that comes through our mouths.”
Personal testimonials influence self-care	“I would put myself as an example to them, so they can see how I am. They should take care of themselves on time, so they don’t get to be in the situation I am.”
	“I think just having you showing videos, and having people give their testimonies … how they got to that point.”
Videos, social media and community locations	“They could give information on the TV or … even on Facebook. Most people never put their phones down because they are using Facebook. Maybe they could give a statement at [church]. They regularly make announcements when the mass is over.”
	“I think … at a community place where people gather, at a church [where] a lot of people attend good places. Places where people have a frequency [of] attending.”

### Lack of Kidney Disease Awareness

#### Dialysis Was Unexpected

When asked to recall how they found out about their kidney disease, participants described developing severe symptoms, presenting acutely ill, and then being informed their kidneys were not working and they needed to start dialysis. This news came as a surprise, because they previously were unaware of any kidney problems or were only recently informed.

#### Missed Opportunity for Prevention

Participants largely blamed lifestyle behaviors and lack of knowledge for their kidney failure. They expressed remorse over not knowing the importance of nutrition, and wished they had known how to prevent their kidney failure. They felt if they had known what they needed to change earlier, they might not have needed dialysis.

### Education Provided Was Incomplete and Poor Quality

#### Treatment Options Influenced by Nonmedical Factors

The main treatment option discussed for participants’ kidney failure was emergency-only dialysis. Participants demonstrated little understanding of other options, including in-center hemodialysis, home dialysis modalities, transplantation, and palliative care. They understood their options were restricted because of citizenship and insurance status.

#### Education Not Delivered at Appropriate Level of Health Literacy

Participants experienced confusion during discussions with clinicians. Their limited understanding of kidney disease made it difficult to process what they were told, ask questions, and make decisions. The large volume of information conveyed at one time made it especially difficult to process what was happening. Most participants believed they were in kidney failure because of their nutritional habits, but did not demonstrate a clear understanding of what caused their kidney disease or how it could have been prevented.

#### Education Provided While Experiencing Distress

Participants reported they often felt too ill to engage in discussions with clinicians. Since most education happened while they were experiencing symptoms of volume overload and uremia, they felt too sick to understand, inquire about options, or make decisions. They reported being only interested in feeling better, not in understanding what was happening to their bodies, and would have accepted any treatment option without resistance.

### Lack of Culturally Concordant Communication and Care

#### Education Did Not Occur in Preferred Language

Participants reported the education they received frequently did not happen in their preferred language. Interpretation services were sometimes used when clinicians did not speak their language, but not consistently. They did not advocate for interpretation services when needed.

#### Education Did Not Incorporate Support System

Participants’ felt their family members and social supports understood that their kidneys were not working, but they had little understanding of what they were experiencing physically and emotionally or what to expect. Participants expressed concern about the impact of their diagnosis on family members, especially children. They felt if their family members knew what was considered normal, it might alleviate some of the panic associated with waiting for them to develop acute indications for dialysis. Educating family members about fluid, sodium, potassium, and phosphorus restrictions might help patients adopt dietary changes.

### Elements Latinx Patients Receiving Emergency-Only Dialysis Want in Their Education

#### Give Overall Prognosis and Describe Expected Symptoms

Participants wanted to know how kidney failure and dialysis would affect their everyday lives, how long they were expected to live, and whether they should have hope for recovery or change. They wished they were told what symptoms to expect from kidney failure, and both before, during, and after dialysis. Understanding the expected variety of symptoms and their trajectory would have normalized their experience and alleviated anxiety.

#### Use Culturally Concordant Language

Participants preferred to receive news and education in their native language by native speakers. Hearing news and having discussions with native speakers would have facilitated greater understanding, and provided an environment that enabled them to express preferences and ask questions.

#### Use Visual Aids

Some topics, even if explained in the participants’ native language, were difficult to understand. Participants reported that seeing pictures of central venous catheters or arteriovenous fistulas would have improved their understanding before they underwent vascular procedures.

#### Incorporate Traditional Foods into Dietary Counseling

Participants made several changes to their diet after starting dialysis. They demonstrated knowledge about the importance potassium and fluid intake, how dietary factors affected their need for dialysis, and that they were able to delay treatments through avoidance. Understanding which culturally traditional foods they needed to avoid or were safe to eat was helpful, including desserts, produce (ie, cactus), and tortillas.

### Facilitators of Patient Activation and Coping

#### Encouragement and Normalization of Emotional Experience

Participants experienced depressed mood and grief about their diagnosis, and about the life changes required of them to undergo emergency-only dialysis. They expressed frustration about the inability to work, and the fact that their lives revolved around hospital admissions. It would have helped them to know that symptoms of depression and anxiety were common. They appreciated encouraging messages from medical staff, and wanted clinicians to inform family members to encourage them as well.

#### Include Family in Decision-Making

Participants highlighted the importance of involving their family and support systems in their care. They wanted family members to understand the diagnosis, what they would experience physically and emotionally, and what treatment would feel like for the patient. Living for family motivated participants to continue receiving dialysis, despite the emotional and physical distress it entailed. Most participants reported they were not doing dialysis for themselves, but for their families.

#### Faith

Spirituality and religion motivated participants when they were feeling ill or depressed. Despite the hardship they were experiencing, spirituality enabled participants to explain what they could not otherwise understand.

### Latinx Patient Recommendations to Improve Community Outreach

#### Promote Diet and Lifestyle Education

Participants felt their community needed a better understanding of how to eat and incorporate healthy behaviors like physical activity. They acknowledged it was common to believe one did not need to engage in healthy behaviors unless they had a medical condition. Bonding over meals is an important cultural tradition, so highlighting the need for universal healthy eating practices might be an opportunity to reach numerous people.

#### Personal Testimonials Influence Self-Care

Participants felt the best way to help their community understand the importance of kidney health was by telling their personal stories about dialysis. If others understood what happened to them and what it was like to have kidney failure, they might be motivated to make changes or be proactive about their medical care.

#### Videos, Social Media, and Community Locations

When asked where community outreach should occur, participants suggested places where many individuals frequent and feel safe, such as churches, schools, and the consulates. Videos were preferred over written materials, and they believed using television, soap operas, and social media would have higher impact.

## Discussion

Latinx adults who rely on emergency-only dialysis receive fragmented and substandard care, and little is known about their kidney disease education or their perspectives on how education and outreach might be improved. Participants often were unaware they had kidney disease until they had severe or end-stage disease. In some cases, they learned of their diagnosis when they initiated emergent dialysis. The education they received was poor quality, and often not culturally tailored. Participants wanted to know their overall prognosis, what they will experience both physically and emotionally with dialysis, and they wanted their families involved in education and decision making. Use of patient testimonials and nontraditional platforms such as social media and classes in community locations may optimize the impact of community outreach efforts. Our findings have important implications for clinical practice (Figure).

**Figure.**
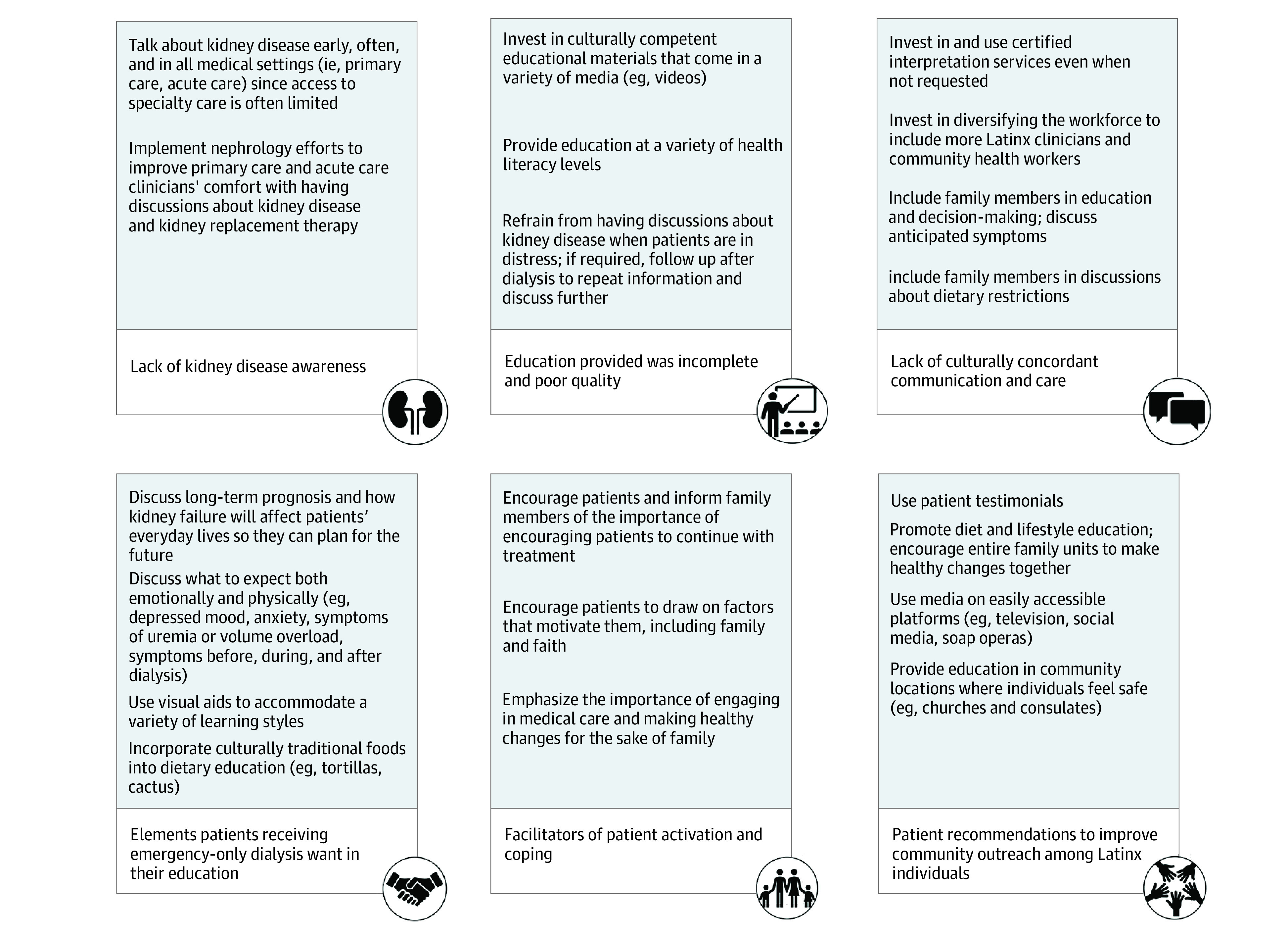
Themes With Associated Implications for Clinical Practice

To our knowledge, this is the first study that describes Latinx undocumented immigrant perspectives on the kidney disease education. Our findings are similar to past studies that document low kidney disease awareness in the Latinx population, which has historically been underserved by the health care system.^18,19,20^ In a cross-sectional study evaluating predictors of kidney disease awareness among people with chronic kidney disease in the National Health and Nutrition Examination Survey, 65% of those without kidney disease awareness spoke Spanish as their primary language, compared with 28% of those with kidney disease awareness.^20^ Our findings are also similar to past studies that document the importance of family and social supports in this population, the need to involve family in disease education and decision making,^3,4,21,22,23,24^ and the role of spirituality in coping.^25^

Tackling kidney disease awareness in Latinx populations is of upmost importance. For the general population, the bulk of education about kidney disease and kidney replacement therapy occurs in nephrology offices and at outpatient dialysis facilities. Because the Latinx undocumented immigrant population does not have consistent access to traditional services, increased education is needed in both primary care and acute care settings.^3,23,26,27,28^ When possible, patients need to be informed they have kidney disease, what that means, how they can slow progression, and the possibility of someday needing kidney replacement therapy. Having these discussions early-on and often, in a language they understand, is the first step in alleviating some of the shock and confusion they experience if dialysis comes as a surprise. Nephrology efforts to improve primary care and acute care clinicians’ comfort with having discussions about advanced kidney disease and kidney replacement therapy should be implemented to facilitate education efforts.

We found that providing patient education in Spanish by native speakers would have facilitated greater understanding of kidney disease and the dialysis process. Providing information in Spanish to patients with limited English proficiency is required by Title VI of the Civil Rights Act, yet many of our participants did not get the information they needed in Spanish.^29^ This highlights the importance of investing in formal interpretation services and using them even if patients do not request them. Diversifying the health care workforce to include more Latinx clinicians is needed. Efforts should also include investment in community health workers, members of the community that have been shown to improve outcomes among Latinx populations.^23,30,31,32^

Our findings suggest that educating the patients’ support system about kidney failure is nearly as important as educating the patient for Latinx individuals. Participants felt their family members had limited understanding of what they were experiencing, and concern about how their illness affected family members added to their stress. If their family understood what they experienced before, during and after dialysis, that might alleviate distress and help family members provide the support they needed. Incorporating family members in the education and decision-making process may also promote patient understanding when they feel too ill to engage with clinicians.

Patient activation is historically low among Latinx populations,^33,34,35^ and efforts to improve patient activation and engagement in care are critical to prevent kidney failure. We found that participants were motivated when they were encouraged and felt supported by the medical staff, and when they focused on working hard, exhibiting strength for the sake of their families, and drawing on their faith. Emphasizing facilitators during community outreach may also encourage earlier incorporation of healthy lifestyle behaviors. For example, individuals might be more willing to make healthy dietary changes early in the course of their kidney disease if they feel they are doing it for their family, or if family members are also making changes.

Increasing access to primary care, nephrology care, and scheduled outpatient dialysis for immigrant populations is essential. Some states have increased access to primary and specialty care by expanding Medicaid benefits to undocumented immigrants.^8,36^ In 12 states, undocumented immigrants with kidney failure are able to receive scheduled outpatient dialysis. This was accomplished by changing the scope of each states’ Emergency Medicaid scope, so that outpatient dialysis was covered in addition to emergency-only dialysis in the inpatient setting.^7,37^ Not only would this enable access to the kidney failure education provided at outpatient dialysis clinics, but paying for scheduled dialysis would potentially save $5768 per person per month and improve morbidity, mortality, mental health symptoms, and quality of life.^1,2,3,7,38^

### Limitations

Some limitations bear mention. This study used convenience sampling, and the majority of participants were Mexican. The Latinx population is heterogeneous, and we may not have captured themes or experiences of Latinx individuals from other countries. Mexican Americans make up the largest Latinx group in the United States, among whom the age-adjusted prevalence of chronic kidney disease has increased over the past 2 decades, making our findings highly relavent.^28,39^ The education provided may differ in other cities and states, especially those that offer more treatment options for this population. However, our findings are applicable even in states that cover scheduled outpatient dialysis for undocumented immigrants, because Latinx populations continue to face barriers to primary and nephrology care across the country.^9^

Social desirability bias may have caused some participants to censor negative experiences. Our interviewers were bicultural, and used techniques to facilitate rapport and comfort during the interview. We did not assess specific educational materials. Although we cannot generalize findings to broader groups, our recommendations may help Latinx immigrants regardless of their documentation status.

## Conclusions

This qualitative study found that Latinx adults who received emergency-only dialysis received disjointed medical care. The education they received about kidney disease frequently happened too late, was not culturally tailored, and was often poor quality. Describing overall prognosis and what to expect physically and emotionally is preferred, and using culturally concordant language with the assistance of visual aids when needed would facilitate understanding. Using patient testimonials and providing education on television, social media and at community locations might improve the breadth and impact of outreach efforts. Messaging that incorporates ideals that are important for this population, such as work ethic, strength for the sake of family, and faith, may motivate patients to engage in care, incorporate healthy behaviors, and continue receiving dialysis despite physical and emotional distress.
